# The usage and advantages of several common amyotrophic lateral sclerosis animal models

**DOI:** 10.3389/fnins.2024.1341109

**Published:** 2024-02-26

**Authors:** Lijun Zhou, Meng Xie, Xinxin Wang, Renshi Xu

**Affiliations:** ^1^Department of Neurology, Jiangxi Provincial People’s Hospital, Clinical College of Nanchang Medical College, First Affiliated Hospital of Nanchang Medical College, National Regional Center for Neurological Diseases, Xiangya Hospital of Central South University Jiangxi Hospital, Nanchang, Jiangxi, China; ^2^Medical College of Nanchang University, Nanchang, Jiangxi, China; ^3^Health Management Center, Jiangxi Provincial People’s Hospital, The First Affiliated Hospital of Nanchang Medical College, The Clinical College of Nanchang Medical College, Nanchang, Jiangxi, China

**Keywords:** amyotrophic lateral sclerosis, animal models, motor neuron degeneration, pathology, pathogenesis

## Abstract

Amyotrophic lateral sclerosis is a fatal, multigenic, multifactorial neurodegenerative disease characterized by upper and lower motor neuron loss. Animal models are essential for investigating pathogenesis and reflecting clinical manifestations, particularly in developing reasonable prevention and therapeutic methods for human diseases. Over the decades, researchers have established a host of different animal models in order to dissect amyotrophic lateral sclerosis (ALS), such as yeast, worms, flies, zebrafish, mice, rats, pigs, dogs, and more recently, non-human primates. Although these models show different peculiarities, they are all useful and complementary to dissect the pathological mechanisms of motor neuron degeneration in ALS, contributing to the development of new promising therapeutics. In this review, we describe several common animal models in ALS, classified by the naturally occurring and experimentally induced, pointing out their features in modeling, the onset and progression of the pathology, and their specific pathological hallmarks. Moreover, we highlight the pros and cons aimed at helping the researcher select the most appropriate among those common experimental animal models when designing a preclinical ALS study.

## 1 Introduction

**Figure 1:**
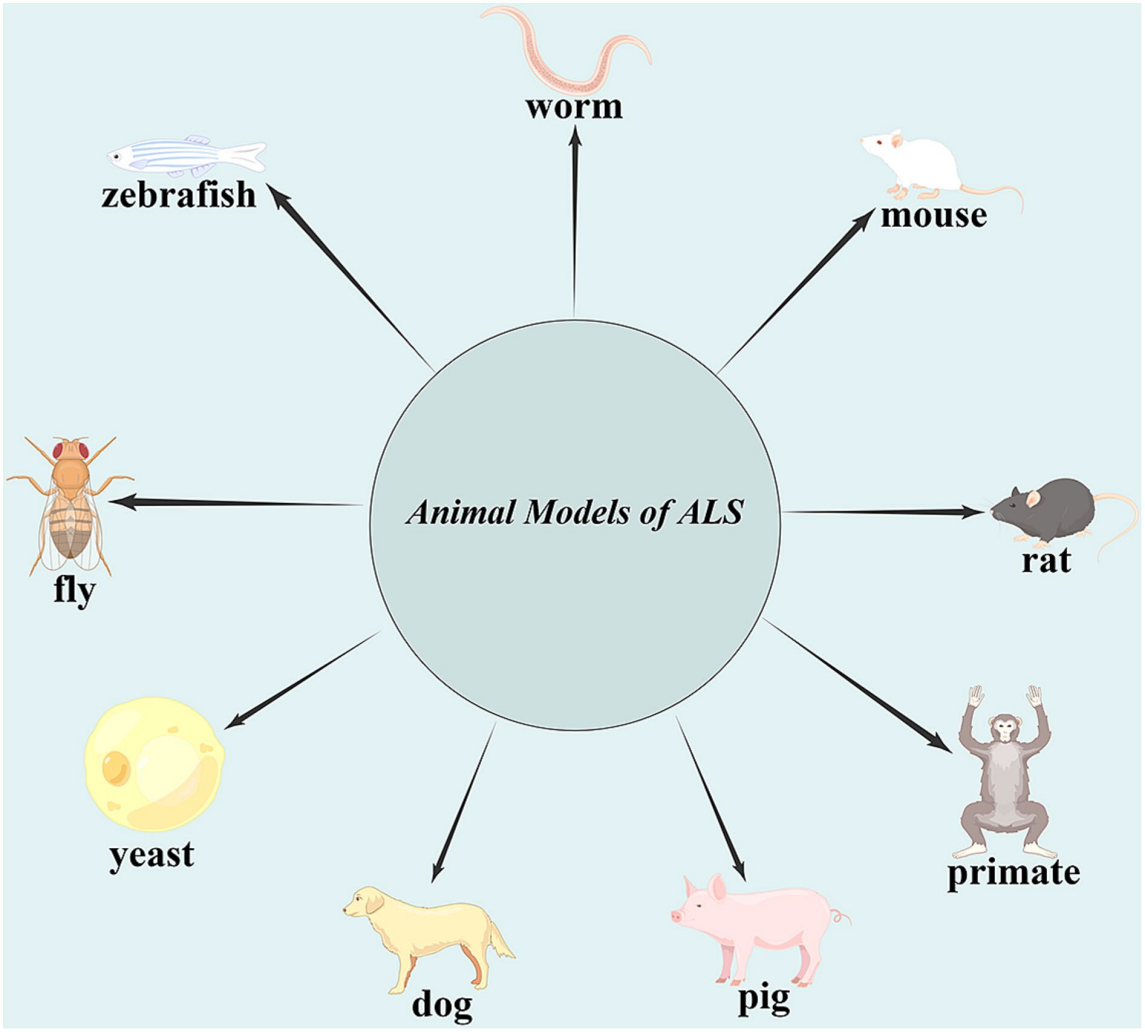
Animal models of amyotrophic lateral sclerosis (ALS). The identification of gene abnormalities associated with ALS led to the development of yeast, worms, flies, zebrafish, mice, rats, pigs, dogs, and, more recently, non-human primate ALS models, which mimic many, but not all, aspects of the human condition.

Amyotrophic lateral sclerosis (ALS) is the most common type of motor neuron disease characterized by damage to upper and lower motor neurons, leading to gradual muscle weakness and muscle atrophy, and finally death due to respiratory failure after 3–5 years of onset. Approximately 90% of ALS cases have unknown etiology and are called sporadic amyotrophic lateral sclerosis (sALS). Approximately 5–10% of ALS patients have a family genetic history called familial ALS (fALS). So far, the etiology and pathogenesis of ALS are not well understood, and there is still a lack of effective treatment. In order to further explore the etiology and pathogenesis of ALS and establish reasonable prevention and control measures, it is important to establish animal models that can reflect the clinical manifestations and pathological characteristics of ALS (Figure 1). This review summarizes the current situation of ALS animal models (Table 1).

**Table 1:** Pros and cons of several common ALS animal models.

Groups	Animal models	Phenotypes and Pathology	Pros and Cons
Naturally occurring animal models in ALS	The wobbler mouse	Autosomal recessive inheritance, mapped to chromosome 11, responsible gene is unknown.	Clinical processes and pathological states closer to human motor neuron diseases.
		The gradual weakness of forelimbs after 3–4 weeks of birth, the brain is rarely damaged, can grow into adulthood, die in about 1 year.	Suitable to study the pathogenesis of neuronal degeneration in ALS disease and the mechanism of antioxidant therapy.
		The selective damage of motor neurons in spinal cord.	
	Progressive motor neuropathy (PMN) mouse	Autosomal recessive inheritance, mapped to chromosome 13, responsible gene is unknown.	Gradual progression of motor neuron loss observed in human ALS.
		The weakness of the pelvic muscles and hind limbs at 3 weeks after birth, and death occurs at the age of 6–7 weeks.	
		The obvious distal motor axonal disease with relatively unaffected the somatic motor neurons.	
	Motor neuron degeneration (MND) mouse	Autosomal dominant inheritance, mapped to chromosome 8, responsible gene is unknown.	Involvement far beyond the motor system of the central nervous system.
		The stiffness, atrophy, and paralysis of hindlimbs at the age of 5–11 months, the survival time rarely exceeds 14 months.	
		The motor neuron degeneration in spinal cord, the hypoglossal nucleus and the motor cortex degeneration.	Can lead to severe retinal degeneration.
	Neuromuscular degeneration (NMD) mouse	Autosomal recessive inheritance, mapped to chromosome 19, responsible gene is SMbp2.	Rapid progression of motor neuron loss observed in human ALS.
		The rapid progression of hindlimb weakness at 2 weeks of age, the survival time rarely exceeds 4 weeks.	
		The degeneration of lumbar motor neuron somas.	
	Hereditary canine spinal muscular atrophy (HCSMA)	Autosomal dominant inheritance.	Similar to ALS disease in humans, the extraocular muscle movements are normal.
		The initial symptoms appear from 4 weeks to 4–6 months of age, manifested as the muscle weakness and the proximal muscle atrophy.	
		The spherical inclusion bodies of nerve microfilaments in the cytoplasm of motor neurons.	
Experimentally induced animal models in ALS	Animal models of neurotoxicity	Aluminum poisoning model, neurotoxin of beta-N-methylamino-L-alanine model.	Clinically delaying disease progression can be achieved by intervening these neurotoxic reactions.
		Various neurotoxicities can produce chronic neuronal damage, leads to some slowly progressing neurodegenerative diseases.	Study the pathological mechanism of abnormalities in the metabolism of various metal ions and excitatory amino acid in the patients with ALS.
	Immune-mediated animal models	Inject antigen or antibody into the models to produce the immune response.	Provides a good research tool for the immunotherapy and vaccine research of ALS.
		Elucidate the mechanism of immune-mediated neuronal damage from both exogenous antigens and antibodies.	
	Transgenic animal models	SOD1 gene mutations: overexpression of G93A/ G37R/G86R, and its cytotoxic effects aggravating the damage to nerve cells.	SOD1-G93A mouse model: the most widely used in experimental animal model of ALS, for researching the mechanism of toxic free radicals in the pathogenesis of fALS and exploring new therapeutic methods.
		Apoptosis gene mutations: the SOD1-G93 transgenic mouse overexpression of BCL-2 or ICE, delaying ALS onset and prolonging its survival.	Apoptosis: only a way of neuronal loss, and can only provide a reference for investigating ALS treatment.
		Inclusion body gene mutations: the lewy hyaline inclusion bodies were found in the cytoplasm of motor neurons from the SOD1-G93/A4V transgenic mouse, may leading to the death of neurons.	Inclusion body: its mutations causing the onset of fALS patients.
		Neural microfilament gene mutations: the overexpressing NF-L/NF-H transgenic mice exhibited a progressive aggravation of motor weakness and muscleatrophy, its motor neurons filled with the phosphorylated NF.	NF: the absence or reduction of NF within axons may play a role in the pathogenesis of SOD1 mutants.
		Golgi apparatus lesion: may play a crucial role in the pathogenesis of the vast majority of sALS and SOD1 mutants fALS.	Golgi apparatus: is a better model studying.

Pros, Advantages; Cons, Disadvantages.

## 2 Naturally occurring animal models in ALS

There are four types of mouse models and one type of dog model in naturally occurring ALS models.

### 2.1 The wobbler mouse

The wobbler mouse model is the most studied in the ALS models of naturally occurring animals. The recessive inheritance of autosomal mutation chromosome 11 is known to cause the degenerative changes of motor neurons in the spinal cord, which is similar to the neuronal damage and clinical manifestations in ALS disease (Kaupmann et al., 1992), but the responsible gene has not been identified. It is characterized by the gradual weakness of the forelimbs after 3–4 weeks of birth, manifested as walking shaking, front paw weakness, and gradual paralysis of the front paw. Pathology is manifested as the selective damage of motor neurons in the spinal cord, which exhibited proximal axon degeneration and vacuolar changes, decreased endoplasmic reticulum, nuclear enamel, and mitochondrial dysfunction. Neurogenic atrophy of muscles is accompanied by the abnormal metabolism of amino acids and peroxides. Electrophysiological studies have shown denervation in the forelimbs of the wobbler mouse (Andrews et al., 1974). The wobbler mouse can be classified as an axonal disease and a neuron disease, but the brain of the wobbler mouse is rarely damaged, and it can grow into adulthood and die in approximately 1 year. The wobbler mouse model is suitable for studying the pathogenesis of neuronal degeneration in ALS disease because of its characteristics of neuronal degeneration, and it has certain value in studying the mechanism of antioxidant therapy (Dave et al., 2003). Recently, Meyer et al. (2024) discovered that the wobbler mouse model of ALS showed hypercorticoidism and neuroinflammation, which could be subsided by treatment with the glucocorticoid receptor (GR) modulator Dazucorilant (CORT113176), indicating that glucocorticoids are probably involved in neuroinflammation. Thus, the GR modulation would become useful to dampen the inflammatory component of neurodegenerative disorders including ALS.

### 2.2 Progressive motor neuropathy mouse

The progressive motor neuropathy (PMN) mouse is the chromosome 13 autosomal recessive mouse model, and the exact genetic defect is unknown at present (Brunialti et al., 1995). The PMN mouse model is characterized by weakness of the pelvic muscles and hindlimbs at 3 weeks after birth, and death occurs at the age of 6–7 weeks (Schmalbruch et al., 1991). The pathological features are the obvious distal motor axonal disease with relatively unaffected somatic motor neurons, which is similar to the pathological changes of human ALS. Schäfer et al. (2017) studied the PMN mice carrying a missense loss-of-function mutation in tubulin-binding cofactor E, which manifested a particularly aggressive form of motor axon dying back and displayed a microtubule loss, being similar to that induced by human ALS-linked tubulin-alpha4a mutations. They unraveled sensory neuropathy as a pathological feature of the PMN mouse and discussed the potential contribution of cytoskeletal defects to sensory neuropathy in human motor neuron diseases including ALS.

### 2.3 Motor neuron degeneration mouse

Motor neuron degeneration (MND) mouse is an autosomal dominant mouse model mapped to chromosome 8, and the defective gene is unknown to date. The MND mouse model is characterized by the stiffness, atrophy, and paralysis of hindlimbs at the age of 5–11 months, and the survival time rarely exceeds 14 months (Messer and Flaherty, 1986). Pathology is characterized by motor neuron degeneration in the spinal cord and hypoglossal nucleus and motor cortex degeneration, which is featured by neuronal swelling with cytoplasmic inclusion bodies (Messer et al., 1987). Involvement far beyond the motor system of the central nervous system can also lead to severe retinal degeneration (Messer et al., 1992).

### 2.4 Neuromuscular degeneration mouse

Neuromuscular degeneration (NMD) mouse is an autosomal recessive mouse model mapped to chromosome 19. The gene defect is the deletion of a single amino acid and the mutation of the splicing donor site encoding the expression of ATPase/DNA lysis helicase gene, which is known as SMbp2 (Cox et al., 1998), and is characterized by the rapid progression of hindlimbs weakness at 2 weeks of age, and the survival time rarely exceeds 4 weeks. Pathology is characterized by the degeneration of lumbar motor neuron somas (Cook et al., 1995).

### 2.5 Hereditary canine spinal muscular atrophy

Hereditary canine spinal muscular atrophy (HCSMA) is a model of the Spanish hunting dog of Brittany, which is an autosomal dominant inheritance and is divided into three phenotypes: early-onset, intermediate-, and late-onset phenotypes (Cork et al., 1980). According to the phenotype, the initial symptoms appear from 4 weeks to 4–6 months of age, which are manifested as muscle weakness and proximal muscle atrophy. Pathology is characterized by the spherical inclusion bodies of nerve microfilaments in the cytoplasm of motor neurons. The anterior horn of the spinal cord becomes smaller, and the dysfunction of the L-subunit coding gene of the nerve microfilaments leads to a decrease in the diameter of the axons in this region. Except in advanced disease, the number of motor neurons in the anterior horn of the spinal cord is not necessarily reduced. Similar to ALS disease in humans, the extraocular muscle movements are normal. Up to now, only two missense mutations in SOD1 dismutase, T18S, and E40K have been identified as the molecular determinants for HCSMA. Both T18S and E40K mutations induced the SOD1 aggregation, possibly by reducing the negative charge repulsion, disrupting the E40-K41 salt bridge, or forming the disulfide-linked enzymatically active dimers, thus resulting in a SOD1 toxic gain-of-function (Crisp et al., 2013; Kimura et al., 2020) and showing the recessive inheritance with reduced penetrance (Awano et al., 2009; Wininger et al., 2011). Besides the motor neuron loss, canine models affected by HCSMA share some other pathological features with SOD1-ALS rodent models and patients, such as oligodendrocyte injury, leading to demyelination (Golubczyk et al., 2019), increase in arginase 1-expressing microglia in the proximity of motor neurons (Toedebusch et al., 2018), and upregulation of cannabinoid receptor 2 in reactive astrocytes (Fernández-Trapero et al., 2017).

The pervasive and naturally occurring motor weakness exhibited by naturally occurring animal models with more subacute or chronic pathogenesis mimics the apparent spontaneous and gradual progression of motor neuron loss observed in human ALS. The wobbler and MND models exhibit clinical processes and pathological states closer to human motor neuron diseases, but one of the main difficulties with using these mice as experimental models is that we do not know if the conditions of these specific models have similar molecular or biochemical defects to human ALS. This difficulty may change once the genetic and molecular mechanisms of motor neuron loss in these mice are recognized (Elliott, 1999).

## 3 Experimentally induced animal models in ALS

### 3.1 Animal models of neurotoxicity

In 1969, Olney et al. proposed the concept of excitotoxicity (Olney et al., 1986), and since then, more and more evidence has shown that various neurotoxicities can produce chronic neuronal damage, which leads to some slowly progressing neurodegenerative diseases, and clinically delaying disease progression can be achieved by intervening in these neurotoxic reactions.

Aluminum has a chronic toxic effect on neuronal cells. Kihira et al. (1995) copied ALS animal models with the aluminum poisoning method. The repeated injection of aluminum chloride in the occipital large pool of rabbits can produce chronic neurotoxic effects on the motor neuron soma and dendrites in the anterior horn of the lumbar spinal cord, which appears as obvious edema and endoplasmic reticulum rupture, and a large number of nerve microfilaments, free ribosomes, and lipid-like substances are aggregated and generate a wide range of inclusion bodies. The aluminum model is very similar to inclusion bodies, nerve microfilament abnormalities, and axonal transport disorders in ALS patients.

Duncan (1991) reported that the neurotoxins of neurotoxic amino acid beta-N-methylamino-L-alanine in iron trees can produce neurotoxic effects on neurons. The toxic animal model of beta-N-methylamino-L-alanine showed the signs and behavioral abnormalities of ALS patients, and the pathological examination of this model showed the degeneration of motor neurons and chromosomal lysis in the anterior horn of the spinal cord.

These neurotoxic animal models can be used to study the pathological mechanism of abnormalities in the metabolism of various metal ions and excitatory amino acids in patients with ALS.

### 3.2 Immune-mediated animal models

In 1964, Lowenthal et al. found the abnormal bands of immunoglobulins in the serum or cerebrospinal fluid of ALS patients by agarose electrophoresis, and since then, the relationship between ALS and autoimmunity began to be studied successively by researchers.

**Figure 2:**
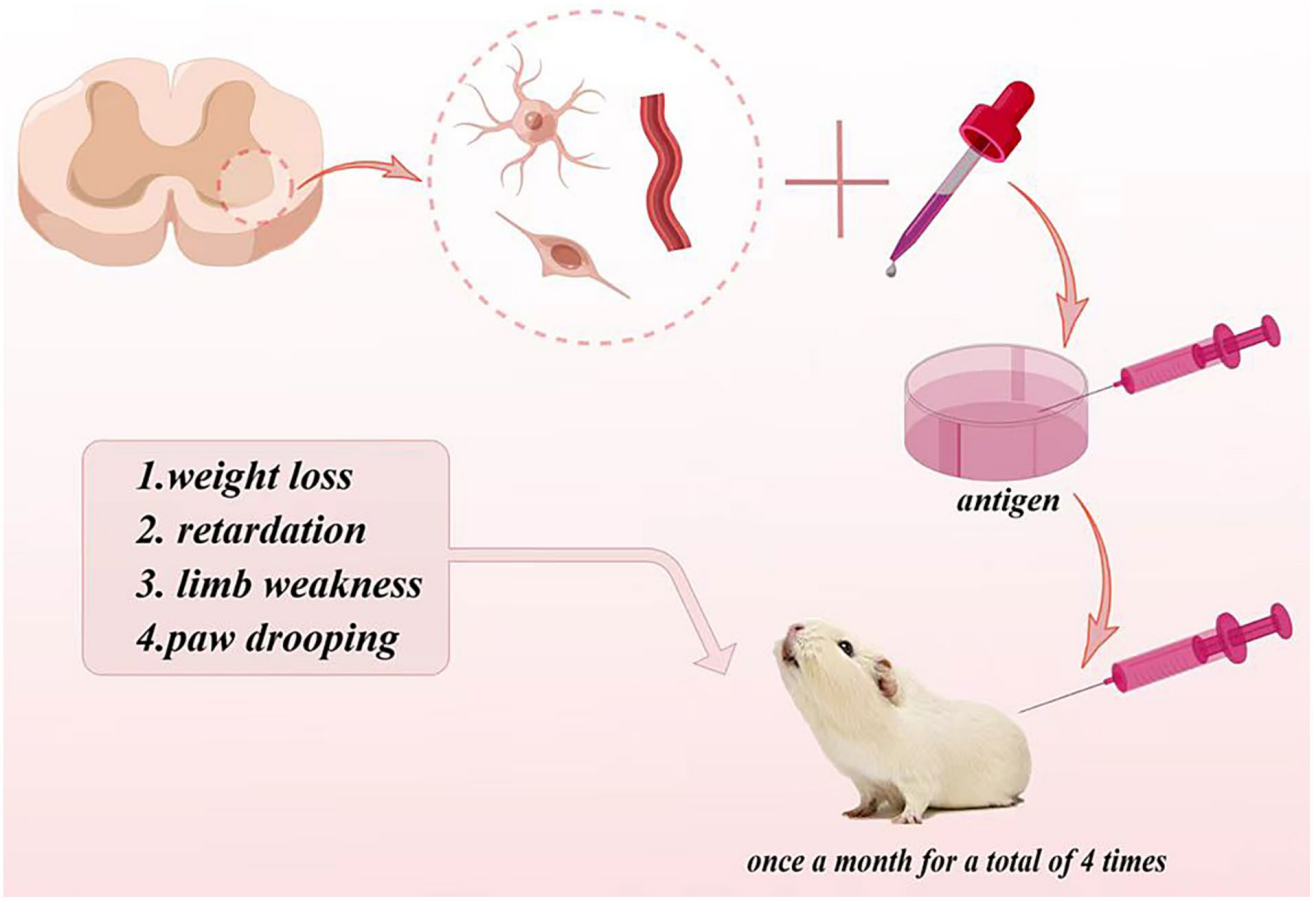
Autoimmune ALS animal model in guinea pigs.

Engelhardt et al. (1990) established the first autoimmune ALS animal model in guinea pig. They isolated the gray matter consisting of nerve cells, cell protrusions, astrocytes, and some small blood vessel fragments in the anterior horn of the cattle spinal cord, then crushed it with ultrasound to make a homogenate, and added the Fuchsian adjuvant to make an antigen. The antigen was injected into guinea pigs once a month for a total of four times. The results showed that more than half of guinea pigs produced an immune response, which was manifested by weight loss, retardation, limb weakness, and paw drooping (Figure 2). Electromyography exhibited the number decrease of motor units. Pathologically, muscle atrophy and anterior horn neurons of the spinal cord and phagocytosis neurons can be observed, and the scale of pathological damage was related to behavior abnormalities. The immunohistochemical staining displayed the positive IgG staining of the motor neurons in anterior horn and nerve-muscle junctions and demonstrated an IgG deposition.

The monoclonal antibody of anti-anterior horn motor neuron McAb24B0, whose titer is 1:3,000 by the ELISA method, was also injected into the tibial anterior muscle of guinea pigs. The monoclonal antibody McAb24B0 could be taken up by the axon terminal in the anterior horn of the spinal cord; it protruded from the anterior horn of the lumbar spinal cord and transported to the soma of the spinal cord and bound to the corresponding antigen of the soma, resulting in motor neuron necrosis and demyelination of myelinated nerve fiber.

This animal model reproduces the autoimmune response to neurons; the preparation operation of this model is simple and the repeat is good; it elucidates the mechanism of immune-mediated neuronal damage from both exogenous antigens and antibodies and provides a good research tool for immunotherapy and vaccine research of ALS. Whether this animal model can truly reflect the autoimmune response pathogenesis of ALS needs to be further studied.

### 3.3 Transgenic animal models

Transgenic animals are genetically engineered animal models, and with the development of transgenic technology and gene mapping technology, the research on transgenic animal models of human genetic diseases has been widely used. The transgenic animal models mainly include both transgenetic and knockout animal models. Transgenetic animal models are applied to the dominant hereditary diseases, and the disease-causing gene is introduced into normal animal cells to produce the corresponding transgenic animal model. Gene knockout animal models are applied to recessive genetic diseases, and the transgenic animal model of the disease is established by knockouting the allele of the disease-causing gene (Rülicke, 1996). A Cre/loxP site-specific recombination system was used to establish a transgenic model (Plück, 1996).

#### 3.3.1 Transgenic animal models of SOD1 gene mutations

The most common genetic form of fALS is the autosomal dominant inheritance. Among them, the Cu-Zn superoxidase (SOD1) gene mutation located on autosome 21 is in the form of point mutation and small deletion, which is found in approximately 25% of fALS patients (Takehisa et al., 2001). SOD1 is Cu^2+^-Zn^2+^-SOD in the cytoplasm, which appears in all cells of aerobic metabolic tissue, and the main function of SOD1 is to resist oxidation and scavenge free radicals. SOD1 is expressed at a high level in neurons with a high degree of conservation. More than 90 types of mutations were found in the SOD1 gene of fALS patients. The common mutation sites are alanine mutates valine at the codon position 4, glycine mutates alanine at the codon position 93, G mutation at the codon position 100, the isoleucine mutates threonine at the codon position 113, and TGGG is inserted into the codon position 127 (Dal Canto and Gurney, 1995).

**Figure 3:**
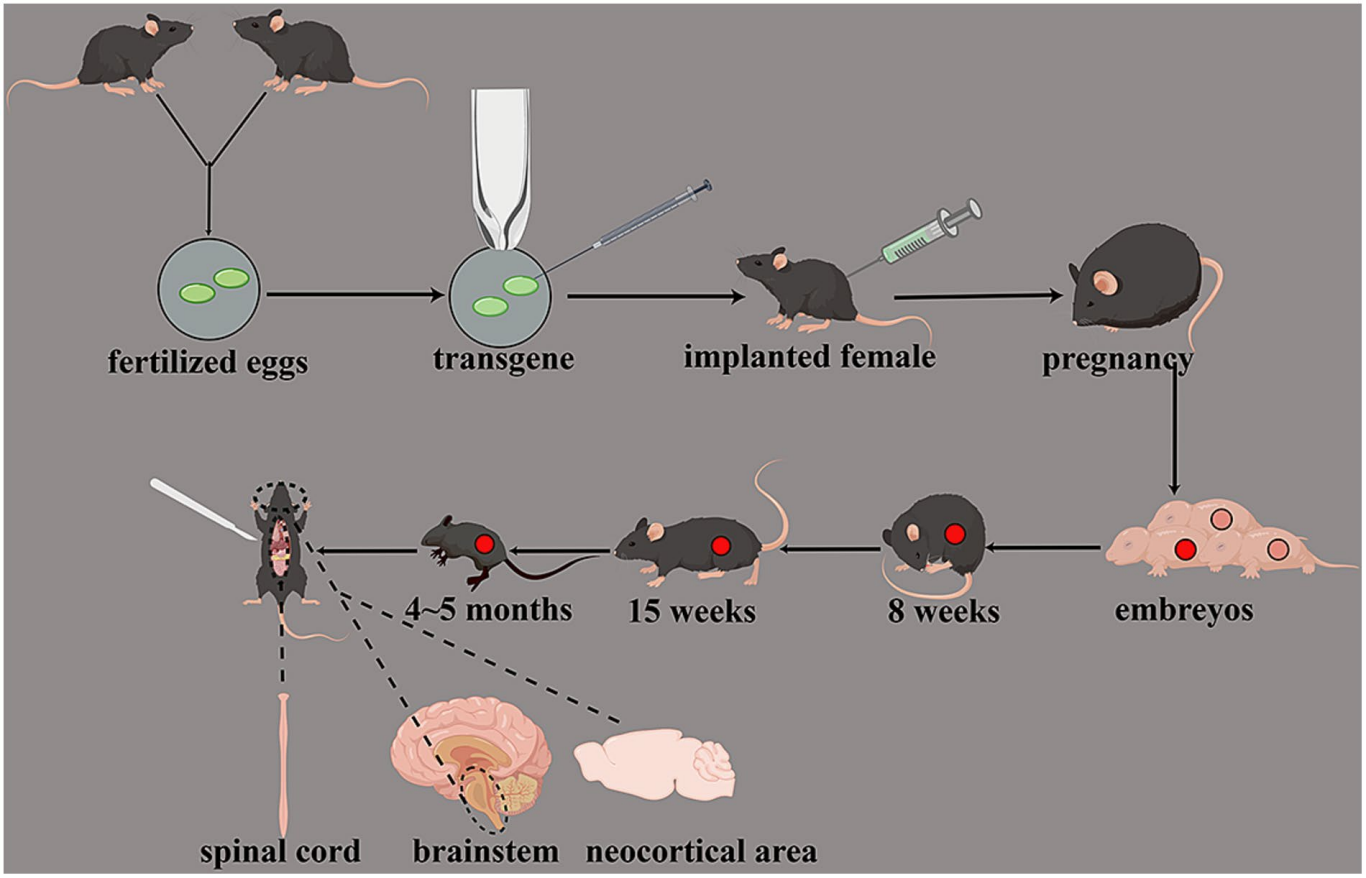
Transgenic animal models of SOD1 gene mutations. Transgenic mice had weakened muscle strength and motor incoordination at approximately 8 weeks; the synaptic activity was damaged at approximately 15 weeks, and death was at approximately 4–5 months. The degeneration of motor neurons in the spinal cord, brainstem, and neocortical area can be observed.

Different kinds of SOD1 gene mutations have different experimental cycles. Gene mutations with a high copy number develop a rapid course and short cycle. Conversely, mutations with a low copy number have a slow progression and are more similar to the onset of ALS in humans. The earliest symptoms of SOD1 G93A transgenic animals were weakened muscle strength, motor incoordination at approximately 8 weeks, synaptic activity damage at approximately 15 weeks, and death at approximately 4–5 months. Gurney (2000) transferred the SOD1 mutated gene of human fALS into mice, and these transgenic mice began to show symptoms similar to human ALS at 3–4 months. The pathology of this mouse model showed a motor neuron deletion in the spinal cord, which is similar to the pathological characteristics of human ALS. This model confirmed the relationship between mouse motor neuron damage and SOD1 gene mutation; hence, it is a putative model for searching the pathogenesis of motor neuron damage in ALS. Gurney et al. (1994) found that the mouse expressing G93A mutant SOD1 exhibits genotypic and pathological characteristics similar to human ALS, while the mouse overexpressing wild-type SOD1 is normal. In addition, Wong et al. (1995) found that the mouse overexpressing SOD1-G37R mutants also eventually exhibited a severe progressive motor neuron disease. Ripps et al. (1995) found that the transgenic mouse with SOD1-G86R mutants also exhibited degeneration of motor neurons in the spinal cord, brainstem, and neocortical area (Figure 3).

Reaume et al. (1996) found that the SOD1 gene-deletion mouse established by the homologous recombination method showed that the mouse could develop normally, and the motor nerve function loss did not occur in 1 year. However, when its axons began to damage, the loss of motor neurons increased significantly compared with the control group, and the loss of distal axons also increased with age. This model suggested that the SOD1 gene is an important factor in promoting physiological decay or secondary damage of neurons.

Pramatarova et al. (2001) reported that the neuron-specific expression of mutant superoxide dismutase 1 in the transgenic mice does not lead to motor impairment, so mutant SOD1 is not necessary for the pathogenesis of ALS.

The cytotoxic effects caused by SOD1 gene mutations may be related to the instability of the SOD1 enzyme; the mutative SOD1 can accelerate the aggregation of toxicants in the body and may produce the high affinity for nerve cells, thereby aggravating the damage to nerve cells.

At present, the animal model of the human mutant SOD1 gene is the most similar experimental animal model of ALS, among which the hmSOD1-G93A mouse model is the most widely used, especially for researching the mechanism of toxic-free radicals in the pathogenesis of fALS and exploring new therapeutic methods. However, only approximately 5% of patients with ALS have SOD1 mutations, and most of them are present in fALS, which cannot explain the pathogenesis of most sALS in which SOD1 is normally expressed. In addition, this animal model has high requirements for experimental conditions, equipment, and technology and can only be carried out in laboratories with transgenic technology, and with a long cycle and high cost.

#### 3.3.2 Transgenic animal models of TDP-43 gene mutations

TAR DNA-binding protein 43 (TDP-43) is an RNA-binding protein associated with various nucleic acid metabolism processes and RNA stability (Liao et al., 2022). In ALS patients, the abnormal deposition of TDP-43 occurs in the cell nucleus and cytoplasm, forming inclusions. The mutations and abnormal aggregation of TDP-43 are closely linked to the pathogenesis of ALS (Suk and Rousseaux, 2020). Introducing human TDP-43 gene mutations into mice or other model animals can replicate the characteristics of human ALS. To gain a deeper understanding of the TDP-43 role in ALS, scientists have developed transgenic animal models related to TDP-43 (Watkins et al., 2021; Oliveira et al., 2023). These models not only enable researchers to study the function of TDP-43 in the nervous system but also provide an opportunity to understand how the TDP-43 abnormal aggregation leads to neuronal damage and death. These models play a crucial role in unraveling the pathogenesis of ALS and exploring potential therapeutic approaches.

One of the earliest transgenic mouse models used to investigate the association between TDP-43 and ALS is the TDP-43 A315T transgenic mouse. By introducing the human TDP-43 A315T mutant into mice, researchers successfully replicated the features of TDP-43 abnormal aggregation and inclusion body formation. Moreover, these mice exhibited neurological pathological features resembling ALS patients, including neuronal damage and muscle atrophy (Dang et al., 2014). The TDP-43 M337V transgenic mouse model utilized another mutated form of TDP-43, M337V, and successfully mimicked TDP-43-related ALS features, including neurodegeneration and motor dysfunction (Arnold et al., 2013). The rNLS8 transgenic mouse model is driven by hTDP-43ΔNLS, a robust regulator of neurons lacking a nuclear localization signal. This model serves as an ALS-like pathological model (Hur et al., 2022). Recently, the rNLS8 transgenic mouse model has been employed to investigate the precise temporal regulation between TDP-43 and cellular stress and death pathways in ALS (Luan et al., 2023).

Utilizing CRISPR/Cas9 technology, researchers can precisely edit mouse genes, including the TDP-43 gene, to generate more specific transgenic mouse models (Onda-Ohto et al., 2023). This aids in a more in-depth exploration of the specific functions and mechanisms of TDP-43 in ALS. Modern TDP-43 transgenic models not only focus on the early stages of disease onset but also emphasize the development and progression of the disease. This allows researchers to comprehensively understand the dynamic role of TDP-43 in ALS and provides more possibilities for disease intervention.

#### 3.3.3 Transgenic animal models of FUS gene mutations

Fused in Sarcoma (FUS) is a nucleocytoplasmic transport protein, and its mutations are closely associated with the pathogenesis of familial and sporadic ALS (Belzil et al., 2009). Researchers have successfully established the transgenic animal models related to ALS by introducing human FUS gene mutations or overexpressing FUS protein, revealing the association between the FUS abnormal aggregation in the nervous system and ALS pathophysiology (Qiu et al., 2021; Kerk et al., 2022).

By introducing the human FUS gene mutation variant R521C into mice, researchers successfully simulated the characteristics associated with FUS and ALS (Qiu et al., 2021). This model demonstrated pathological features similar to ALS patients, including neuronal damage, motor dysfunction, and muscle atrophy (Qiu et al., 2021). The FUS-P525L transgenic model utilized another mutated form of the FUS gene, P525L, and also exhibited neurological abnormalities and impaired motor function reminiscent of ALS (Kerk et al., 2022).

With the discovery of new FUS mutations, researchers continue to establish several novel transgenic animal models to gain a more comprehensive understanding of how FUS mutations contribute to ALS. These models not only involve the overexpression of mutant forms but may also incorporate precise gene editing technologies such as CRISPR/Cas9 (Zhang et al., 2018). Researchers are dedicated to delving into the interactions between FUS and other proteins such as TDP-43 in the pathogenesis of ALS (Loughlin and Wilce, 2019). By establishing the corresponding transgenic models, they aim to elucidate how these proteins’ abnormal interactions collectively contribute to the development of ALS.

In recent years, there has been a focus on conducting longer-term longitudinal studies on FUS transgenic animal models, aiming to observe the dynamic changes in the disease progression. This approach helps uncover how the pathophysiology related to FUS and ALS evolves over time, providing valuable insights for the early diagnosis and treatment of the disease.

#### 3.3.4 Transgenic animal models of C9orf72 gene mutations

Chromosome 9 open reading frame 72 (C9orf72) gene mutations represent a major genetic factor contributing to both familial and sporadic cases of ALS (Balendra and Isaacs, 2018). The abnormal expansion of repeat sequences within this gene is a characteristic associated with ALS and Frontotemporal Dementia (FTD) (Balendra and Isaacs, 2018). Establishing transgenic animal models related to C9orf72 proves instrumental in delving into its mechanisms, uncovering how this gene mutation triggers neurodegenerative diseases.

By introducing the abnormal G4C2 repeat sequence from the human C9orf72 gene, researchers established the C9orf72-G4C2 repeat transgenic mouse model (Stepto et al., 2014). This model exhibits the pathological features associated with ALS, including neuronal damage, motor dysfunction, and abnormalities in RNA metabolism. Additionally, these model mice display behavioral and cognitive abnormalities resembling FTD (Stepto et al., 2014). Drosophila, or fruit flies, have also been utilized as model organisms to study the impact of C9orf72 gene mutations. By introducing the G4C2 repeat sequence of C9orf72 in fruit flies, researchers observed damage to motor neurons and abnormal motor behavior, aligning with the manifestations of human ALS (Goodman et al., 2019).

Further research is focused on the relationship between C9orf72 and RNA metabolism pathways, including transcription, splicing, and transport. Through the development of transgenic animal models, scientists can deeply investigate the specific roles of these pathways in the development of ALS. Using CRISPR/Cas9 technology, researchers can directly edit the C9orf72 gene in mice or other model organisms to simulate the different mutation types (Meijboom et al., 2022). This precise editing helps to better understand how different mutations impact the pathogenesis of ALS. The latest research is exploring how C9orf72 mutations influence the immune system, particularly the occurrence of neuroinflammation (Masrori et al., 2022). Utilizing transgenic animal models, researchers aim to elucidate the relationship between immune abnormalities and ALS pathology.

#### 3.3.5 Advantages and disadvantages of transgenic mouse models in ALS research

The advantages of SOD1-related ALS models lie in the fact that SOD1 gene mutations are among the earliest-discovered genetic mutations associated with ALS. Consequently, SOD1-related ALS models benefit from a broad research history and literature foundation. Additionally, these models exhibit good reproducibility, and introducing different SOD1 mutations can generate multiple transgenic animal models, enabling researchers to explore the impact of various mutation types on the pathogenesis of ALS. Pathologically, SOD1 models demonstrate pathological features similar to human ALS, such as neuronal damage and motor dysfunction. However, the downside of SOD1-related ALS models is their phenotypic heterogeneity; the different SOD1 mutations may lead to diverse phenotypes, complicating the interpretation of research results. Moreover, despite the strong association between SOD1 mutations and ALS, only a minority of ALS patients have SOD1 mutations, limiting the applicability of the model in exploring the overall mechanisms of ALS.

TAR DNA-binding protein 43 gene mutations are widespread in ALS and other neurodegenerative diseases, making TDP-43-related ALS models better able to cover the diversity of different ALS cases. Additionally, TDP-43 is a major component of protein inclusions, commonly found in ALS patients, enhancing the relevance of TDP-43 models to the pathology observed in actual patients. However, the multitude of TDP-43 mutations and the different forms of aggregates make the models complex, posing challenges in determining specific mechanisms. There may also be some issues with overexpression, and some models simulate the disease by overexpressing TDP-43, which might lead to effects unrelated to the processes under investigation.

Fused in Sarcoma gene mutations are associated with ALS, allowing FUS-related models to encompass various genetic variations. FUS, being a nuclear-cytoplasmic protein, has mutations linked to RNA metabolism and transcriptional regulation, enabling FUS-related models to delve into these processes. However, the clinical phenotype variations resulting from FUS mutations add complexity to model interpretation. Additionally, FUS involvement in multiple cellular processes makes it challenging to focus model studies on specific mechanisms.

Chromosome 9 open reading frame 72 gene expansion is one of the most common genetic reasons for ALS, giving C9orf72-related models greater clinical relevance. C9orf72 mutations are associated with immune system activation, providing valuable research opportunities for studying ALS mechanisms related to immunity and neuroinflammation. However, the mechanisms resulting from C9orf72 mutations involve multiple aspects, including RNA metabolism, immunology, and cytoplasmic mechanics, making model studies relatively complex. Similar to SOD1, C9orf72 mutations exhibit high heterogeneity among different patients, increasing the challenges of research.

In summary, each ALS model has its unique strengths and weaknesses, and researchers typically need to choose the most suitable model based on specific aspects of their research questions. Integrating results from multiple models can provide a more comprehensive understanding of the complex mechanisms underlying ALS.

#### 3.3.6 Animal models of apoptosis

Normal cell renovation is mainly carried out in two ways, which are programmed death (apoptosis) and necrosis. Under physiological conditions, cell proliferation is in balance with apoptosis. BCL-2 is an anti-apoptotic protein. Yamashita et al. (2001) established the SOD1-G93A transgenic mouse overexpressing BCL-2; this mouse model could delay ALS onset and prolong its survival but cannot change the outcome of its morbidity. Friedlander et al. produced an interleukin-1βtransferase (ICE) (MI7Z)/ SOD1-G93A transgenic mouse model using a hybrid technology (Friedlander et al., 1997). It found that the ICE (MI7Z)/G93A transgenic mouse survived significantly longer than the SOD1-G93A mouse model. Pasinelli et al. (1998) found that the mutant SOD1-G93A was expressed in neurons, Caspase-1 was activated, its activity was enhanced, and it triggered the secretion of ICE substrate-preleukin-1 β and apoptosis of neurons. However, apoptosis is only a way of neuronal loss, thereby these animal models are not fit to be used to research the pathogenesis of ALS and can only provide a reference for investigating ALS treatment.

#### 3.3.7 Animal models of inclusion body

Gurney et al. (1994) reported that in the fALS case of SOD1-A4V mutant, the Lewy hyaline inclusion bodies in the cytoplasm of motor neurons were found, and they found that the inclusion body was a strong positive aggregate of SOD1 by immunohistochemical staining. This phenomenon is also seen in the neurons of transgenic mice expressing SOD1-G93A. It is speculated that the formation of this inclusion body may interfere with the function of neuronal cells, leading to the death of neurons and causing the onset of fALS patients.

Bruijn et al. (1998) reported that the SOD1-deficient mice were hybridized with SOD1-G85R mice to produce the SOD1-G85R hybrid mice lacking endogenous SOD1 genes. This mouse model exhibited that a SOD1-positive aggregation was still generated regardless of whether there was endogenous SOD1 expression.

#### 3.3.8 Animal models of neural microfilament gene mutations

The genetic animal models of neurofilament (NF) abnormalities are also a research focus of ALS disease. NF is one of the main cytoskeletal components of nerve cells and is the most common structural protein in cells, consisting of light chain (NF-L), medium chain (NF-M), and heavy chain (NF-H). Three subunits of NF are composed in a ratio of 6:2:1. Since nerve cells are highly differentiated post-mitotic cells, they require a good cytoskeleton to maintain their unique cell morphology and physiological functions. One of the characteristic manifestations of ALS patients is the accumulation of abnormally phosphorylated NF, so the animal models of NF overexpression are often used in studying the pathogenesis of ALS (Morrison et al., 1998).

The NF-L transgenic mice exhibited front and rear limb weakness, progressive aggravation of symptoms, and neurogenic atrophy of muscles. The pathological reduction of motor neurons occurred in the spinal cord. Neurons filled with the phosphorylated NF and the proximal axon swelled, which are similar to changes in ALS patients. Transgenic mice overexpressing NF-H were manifested by tremors, progressive gait abnormalities, abnormal flexion of hind limbs, and pathologically large motor neurons, and posterior ganglion neurons contained a large number of NF aggregation and mitochondrial dysfunction. In mice overexpressing the NF-H gene, the threshold of nerve fibers was reduced, the membrane resting potential and conduction velocity were significantly reduced, the amplitude of the action potential was small, the duration of the action potential was prolonged, the Na^+^ channel was strengthened, and K^+^ channels was weakened, which was consistent with neurological manifestations in ALS patients. These mouse models are also the animal models used for studying the pathogenesis and treatment of ALS (Kriz et al., 2000).

Eyer et al. (1998) found that SOD1-G37R transgenic mice and the transgenic mice overexpressing NF-H/β galactosidase combined protein were hybridized to produce SOD1-G37R/NF-H/β galactosidase-combined protein-expressed transgenic mice, and it was found that NF does not play a key role in SOD1-mediated neuronal degeneration. Williamson et al. (1998) found that the complete loss of NF-L produced a rapid onset of SOD1-G85R-mediated ALS, and this correlation decreased with the increase in NF-M and NF-H subunit levels in the cell using SOD1-G85R mice and NF-L deficient mice hybridization. Couillard-Després et al. (1998) found that the mouse that overexpressed SOD1-G37R and NF-H subunits increased its average lifespan by 65% compared with the mouse that simply expressed SOD1-G37R.

Although the damaged NF can sometimes cause neuropathy, NF is not necessary for SOD1 mutants to mediate the pathogenesis of neurodegenerative diseases, but the absence or reduction of NF within axons may play a role in the pathogenesis of SOD1 mutants.

#### 3.3.9 Animal models of the Golgi apparatus lesion

Stieber et al. conducted a controlled study using the transgenic mouse overexpressing both NF-H and SOD1-G93A genes (Stieber et al., 1998) and found that the Golgi apparatus may play a crucial role in the pathogenesis of the vast majority of sALS and SOD1 mutant fALS. This animal model is a better model for studying the potential effects of the Golgi apparatus lesion in the pathogenesis of ALS.

## 4 Prospects and existing problems

Although many studied models of ALS currently are being developed and produced, none of them can fully mimic the characteristics of human ALS disease, which has become one of the limitations that limit the treatment and pathogenesis investigation of ALS. Therefore, the models that objectively, accurately, and comprehensively reproduce the comprehensive characteristics of the pathology, biochemistry, and pathogenesis of human ALS disease still need multidisciplinary, multi-level, and comprehensive research. Establishing an ideal ALS model is the basis for further research on ALS and is necessary for exploring the pathogenesis of ALS and further investigating the development and application investigation of effective drugs for the treatment of ALS on the basis of ALS pathogenesis.

## Author contributions

LZ: Writing – original draft, Writing – review & editing. MX: Writing – original draft, Writing – review & editing. XW: Writing – original draft, Writing – review & editing. RX: Funding acquisition, Writing – original draft, Writing – review & editing, Conceptualization.
